# Effectiveness and safety of a newly designed partially covered tracheal metallic Y-shaped stent for the treatment of high cervical gastro-tracheal or tracheo-esophageal fistula: A retrospective observational study

**DOI:** 10.1097/MD.0000000000025132

**Published:** 2021-03-19

**Authors:** Dechao Jiao, Yuan Yao, Quanhui Zhang, Ling Wang, Jianzhuang Ren, Xinwei Han

**Affiliations:** Department of Interventional Radiology, The First Affiliated Hospital of Zhengzhou University, Interventional Therapy Institute of Zhengzhou University, Zhengzhou, China.

**Keywords:** gastro-tracheal fistula, interventional radiology, tracheal stent, tracheo-esophageal fistula

## Abstract

The aim of this study was to evaluate the effectiveness and safety of a partially covered metallic tracheal Y-shaped stent for the treatment of high cervical gastro-tracheal fistula (GTF) and tracheo-esophageal fistula (TEF). From January 2017 to January 2019, 16 patients with high cervical GTF and TEF received partially covered metallic Y-shaped stent placement under fluoroscopic guidance. The technical and clinical success rates, incidence of major complications, and survival outcomes were analyzed. Eastern Cooperative Oncology Group (ECOG) score and quality of life (SF-36 questionnaire) were compared pre and post stent placement. Technical and clinical success rates were 100% and 81.3%, respectively. Major complications (severe tumor ingrowth, mucostasis, hyperplastic granulation tissue) occurred in 7/16 (43.8%) patients. ECOG score and 5 of the 8 domains of the SF-36 (physical function, role physical, general health, vitality, social function) were significantly improved at 1 month after treatment (*P* < .01). During the median follow-up period of 8.3 months, 9 patients were alive. Median overall survival was 10.3 months (95% CI, 8.0–12.6). The newly designed partially covered tracheal Y-shaped stent appears to be effective and safe for treatment of high cervical GTF and TEF.

## 1 Introduction

High cervical gastro-tracheal fistula (GTF) and tracheo-esophageal fistula (TEF) are uncommon life-threatening conditions resulting from damage caused by irradiation, ischemia, tumor invasion, infection, or gastric acid corrosion.^[1]^ Patients present with severe irritating cough, aspiration pneumonitis, or even massive bleeding. The ideal treatment is considered to be the placement of an esophageal stent to block the fistula; however, a foreign body sensation, stent displacement, and airway compression are common problems with esophageal stents.^[2]^ Covered tubular tracheal stents have also been used to block the fistula, but they cause an irritating cough and are easily displaced. Previous investigations have shown that a Y-shaped tracheal stent design can reduce the probability of stent displacement and that a fully-covered structure promotes sputum retention, especially in patients with Eastern Cooperative Oncology Group (ECOG) score 3 or 4, who are likely to have decreased cough function.^[3,4]^ Keeping these 2 factors in mind, we developed an integrated Y-shaped stent with a partially covered main branch and have been using it to treat selected patients at our center. The aim of this retrospective study was to evaluate the effectiveness and safety of this newly designed partially covered self-expanding metallic stent (SEMS) for treatment of high cervical GTF or TEF.

## 2 Materials and methods

This retrospective study was approved by the Institutional Review Board of The First Affiliated Hospital of Zhengzhou University. All procedures were carried out in accordance with The Code of Ethics of the World Medical Association (Declaration of Helsinki). Written informed consent was obtained from each patient before treatment.

### 2.1 Patients

From January 2017 to January 2019, 16 patients with high cervical GTF or TEF were treated by placement of the newly designed partially covered tracheal Y-shaped SEMS at our center. Patients were eligible for treatment with this novel stent if they

1. had malignant tumor confirmed by pathology;
2. had GTF or TEF confirmed by chest computed tomography (CT), bronchoscopy, or upper gastrointestinal radiography;
3. had been considered unsuitable for treatment with esophageal or tubular tracheal stent or if treatment had failed; and
4. had normal coagulation function.

All patients were symptomatic; the symptoms included cough (more in the supine position), expectoration of large amounts of purulent sputum, dyspnea, pulmonary infection, fever, and disturbed sleep. The primary diagnosis was esophageal cancer in 14 patients and lung cancer in 2 patients; 14 patients had history of radiation therapy.

### 2.2 Preprocedure investigations

**Figure 1:**
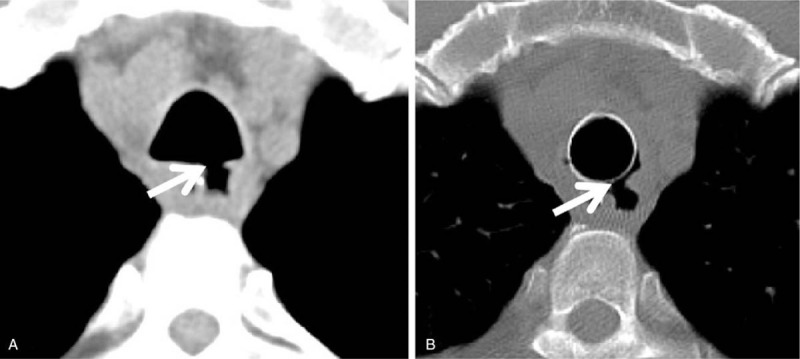
(A): Preoperative computed tomography image shows a high cervical tracheo-esophageal fistula (arrow); (B): the fistula has been completely blocked by the covered stent.

After admission to hospital, all patients underwent blood routine examination, liver and kidney function tests, coagulation function tests, screening for infectious diseases, and electrocardiogram. ECOG and a quality of life (QoL) scale (SF-36 V^TM^; Medical Outcome Trust, Boston, MA, USA) were used to record physical and physiological status. Thoracic spiral CT, with airway reconstruction, or fiberoptic bronchoscopy were performed to clearly visualize the fistula (Fig. 1). Median fistula diameter was 12.8 ± 4.4 mm (range, 5–24 mm). The distance between the fistula and the tracheal carina was >4 cm in all patients. Table 1 lists demographic and clinical characteristics of the patients.

**Table 1:** Demographic and clinical characteristics of the 16 patients.

Patient/Disease characteristic	Value
Age, y, mean ± SD	59.1 ± 8.9 (range: 45.0–72.0)
Sex (male/female)	11 /5
Diagnosis (esophageal cancer/lung cancer)	14 /2
Maximum fistula diameter, mm (median, range)	12.8 (5–24)
Previous treatment (S+I/I/S+C)	10 /4 /2
Clinical manifestation (choking cough/fever)	11/5
Symptom duration	
≥4 wk	9
1–4 wk	5
≤1 wk	2
Diagnostic tools, n (%)	
Chest CT	9
Bronchoscopy + chest CT	7
Final diagnosis (GTF/TEF)	10/6
ECOG score (1/2/3/4)	1 /5 /9 /1
Comorbidity, n (%)	
Hypertension	4
Ischemic heart disease	3
Chronic obstructive pulmonary disease	3
Type 2 diabetes	1

Values are means ± standard deviation. C = chemotherapy, CT = computed tomography, ECOG = Eastern Cooperative Oncology Group, GTF = gastro-tracheal fistula, I = irradiation, S = surgery, SD = standard deviation, TEF = tracheo-esophageal fistula.

### 2.3 Stent design

**Figure 2:**
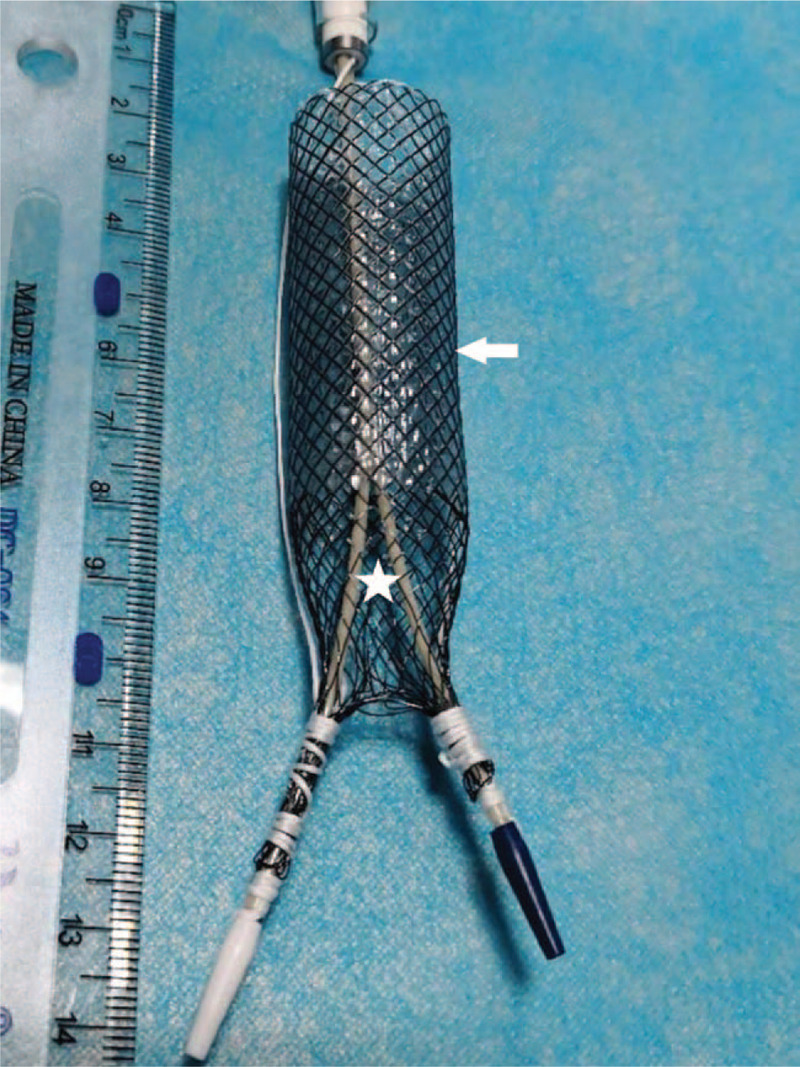
Photograph of the integrated partially covered Y-shaped stent. Covered and non-covered parts are directed by white arrow and star, respectively.

The integrated partially covered Y-shape stent, manufactured by Micro-Tech, Nanjing, China, is woven with a single 0.24 mm nickel–titanium wire; it has 3 parts: a main branch, a left branch, and a right branch (Fig. 2). When placed in the airway, the main branch is located at the lower end of the trachea and the left and right branches in the respective bronchi. The main branch of the stent is partially covered with a silicon membrane, which serves to occlude the opening of the fistula. The length of the entire main branch, the covered part of the main branch, the left branch, and the right branch are 55 to 90 mm, 40 to 60 mm, 15 to 25 mm, and 10 to 20 mm, respectively. The diameters of each branch of the stent are 10% to 15% larger than the corresponding airway (as measured on CT).

### 2.4 Preprocedure treatment

All patients were inserted jejunal nutrition tube through nose, and underwent routine anti-infective, acid suppression, aerosol inhalation, and other support treatments before tracheal stenting.

### 2.5 Stent deployment

For delivery of the stent, the bundle release mode was adopted for the left/right branches and the push release mode for the main branch; the method has been described in detail in our previous report.^[5]^ Subcutaneous diazepam (10 mg) and anisodamine (10 mg), and intravenous dexamethasone (10 mg), were administered 15 minutes before the operation. The patient was placed supine on the digital subtraction angiography examination table, with the head tilted to the right. Mouth gag was placed, and a hydrophilic-coated guide wire (Cook Inc., Bloomington, IN, USA) and a single-bend catheter (Cook Inc., USA) were together passed through the mouth and into the trachea under fluoroscopy. Then, 2% lidocaine and 3 ml of water-soluble iodine were injected through catheter for tracheal mucosal local anesthesia and tracheography. The guide wire (along with the catheter) was manipulated into the left lower lobe bronchus and then exchanged for a super-stiff guide wire (Cook Inc., USA) to establish the left track.

**Figure 3:**
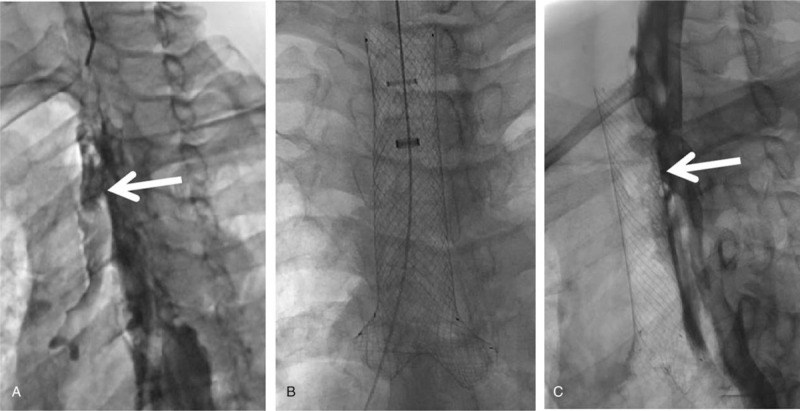
(A): Esophagography after instillation of contrast through the catheter shows a high cervical tracheo-esophageal fistula (arrow); (B): deployment of the Y-shaped stent (main branch 90 mm, with 50 mm covered by silicon membrane); (C): contrast medium esophagography shows the fistula fully blocked by the covered part of the stent (arrow). The Arrow refers to the part covered with plastic film; the 5 stars refers to the part not covered with plastic film.

Another guide wire (Boston Scientific Inc., Natick, MA, USA) was inserted into the right lower lobe bronchus to establish the right track by the same method. The Y-shape stent and the conveying system were mounted over both guide wires and pushed into the main bronchus, rotating the conveying system to make both guide wires parallel within the main bronchus. The push rod was fixed in place, and the outer sheath was pulled back to fully expose the left and right branch. Then, with the push rod and outer sheath fixed, the conveying system was pushed till the 2 branches of the stent were within the respective bronchi. The binding line was pulled to fully release the left and right branches of the stent and then, with the push rod fixed, the outer sheath was pulled back completely to release the main branch of the Y-shaped stent (Fig. 3). Finally, the conveying system was slowly withdrawn. Esophagography, chest spiral CT scan, or tracheoscopy were performed to confirm successful occlusion of the fistula. The patient was returned to the ward, where nebulized budesonide was administered 2 to 3 times a day to alleviate stenting-induced inflammation. Anti-infective measures, mucolytics, and symptomatic treatment were continued (Table 2).

**Table 2:** Procedure parameters and post procedure outcomes.

Data	Value
Stent size, mm, diameter × length, range)	
Covered stent length	40–60
Main tracheal size	18–24 × 55–90
Left branch size	10–14 × 15–25
Right branch size	10–14 × 10–20
Procedure time, min, mean ± SD (range)	42.1 ± 13.3 (22.1–69.2)
Exposure time, min, mean ± SD (range)	18.8 ± 6.3 (5.8–30.1)
Technical success, n (%)	16 (100)
Clinical success, n (%)	13 (81.3)
Post operation ECOG score (0/1/2/3/4)	1/4/9/2/0
Major complications	7
Severe tumor ingrowth	2
Severe mucostasis	1
Severe granulomatous hyperplasia	1
Incomplete closure of fistula	1
Stent intolerance	1
Stent rupture	1
Follow-up treatments (ET/SR/brachytherapy/chemotherapy)	12 /6 /2 /1
Survival outcome (dead/alive)	7/9
Cause of death (severe pneumonia/cachexia/massive hematemesis)	4 /2 /1
Follow-up time, mo, median (95% CI) mo)	8.3 (2.3–18.0)
Overall survival time [mo, median (95% CI), mo]	10.3 (8.0–12.6)
3/6/ 9/12-mo survival rate (%)	93.3/79.4/61.8/41.2

Values are means ± standard deviation or n (%). ET = endoscopic treatment, mo = month, SD = standard deviation, SR = stent replacement.

### 2.6 Follow-up

Technical success was defined as successful placement of the Y-shaped tracheal SEMS, with complete occlusion of the fistula confirmed on CT, tracheoscopy, or esophagography. Clinical success was defined as restoration of the patient's ability to have a semi-liquid diet. ECOG score and QoL (SF-36 V^TM^) were recorded at 1 month after treatment. Major complications were defined as those requiring stent removal, for example, stent displacement, stent intolerance, stent rupture, sputum retention leading to respiratory failure, and progressive granulation tissue hyperplasia or tumor ingrowth despite endoscopic treatments. Minor complications were defined as those not requiring intervention, for example, irritating cough, mild granulation tissue hyperplasia or tumor growth, mild airway mucosal bleeding, and so on. Patients were advised full check-up in hospital at 1 and 2 months after treatment, and every 2 months thereafter. Stent-related complications, follow-up treatments, and deaths were recorded.

### 2.7 Statistical analysis

Data were summarized as median or means ± standard deviation; the pre and poststenting values were compared using the paired t test. Categorical data (ECOG scores) were summarized as percentages and compared using the Fisher exact test. Survival rate was analyzed by the Kaplan–Meier method. Statistical significance was at *P* ≤ .05. SPSS 12.0 (SPSS Inc., Chicago, IL) was used for statistical analysis.

## 3 Results

The technical success rate was 100%. After stent placement, 13 patients were able to consume a semiliquid diet; 1 patient had incomplete fistula occlusion and 2 patients had epiglottic dysfunction. Thus, the clinical success rate was 81.3% (13/16). The mean procedure time and radiation exposure time were 42.1 minutes and 18.8 minutes, respectively. ECOG scores of 0, 1, 2, 3, and 4 were seen in 0, 1, 5, 9, and 1 patient, respectively, before stenting vs 1, 4, 9, 2, and 0 patients, respectively, after stenting; the change was statistically significant (χ^2^ = 9.004, *P* = .030. QoL-related SF-36 domains such as physical function, role physical, general health, vitality, and social function were significantly improved at 1 month after the procedure (*P* < .01); the domains of bodily pain, role emotion, and mental health showed no significant change (*P* = .663, *P* = .083, and *P* = .145; Table 3).

**Table 3:** Scores for quality of life parameters (SF-36) before stenting and at 1 month after stenting.

Parameter	Preprocedure score	Postprocedure score	*t* value	*P*
Physical function	42.8 ± 5.8	58.1 ± 8.3	−5.88	<.001
Role physical	46.9 ± 17.9	64.1 ± 15.7	−3.90	.001
Bodily pain	31.6 ± 8.1	32.9 ± 7.9	−0.44	.663
General health	47.5 ± 8.4	67.7 ± 8.0	−8.11	<.001
Vitality	49.7 ± 8.5	60.6 ± 4.0	−5.47	<.001
Social function	43.8 ± 12.1	58.3 ± 10.5	−5.05	<.001
Role emotion	47.9 ± 17.1	54.1 ± 16.7	−1.86	.083
Mental health	50.8 ± 7.3	53.8 ± 8.0	−1.54	.145

SF-36: Health-related questionare short form-36 were used to assess the 8 patient domains and every domain is calculated into 0–100 scale.

Major complications occurred in 7/16 (43.8%) patients: severe tumor ingrowth in 2 patients, and severe mucostasis, granulation tissue hyperplasia, incomplete closure of fistula, stent intolerance, and stent rupture in 1 patient each. After multiple endoscopic treatments, 4 of these patients underwent stent replacement (at 45, 56 70, and 175 days after the first treatment); 2 patients refused stent replacement after stent removal and instead accepted jejunal nutrition tube placement; 1 patient refused all further treatment after undergoing 2 endoscopic treatments.

Over median follow-up of 8.3 months (95% CI, 2.3–18 months), 7 patients died; the causes of death were severe pneumonia (n = 4), cachexia (n = 2), and massive hematemesis (n = 1). Pretreatment ECOG score was 3 in 9 (56.3%) of these patients. Median overall survival was 10.3 months (95% CI 8.0–12.6). The survival rates at the end of 3, 6, 9, and 12 months were 93.3%, 79.4%, 61.8%, and 41.2%, respectively.

## 4 Discussion

GTF and TEF develop in 5% to 15% patients with malignant esophageal carcinoma and in <1% patients with lung cancers. Patients do not survive beyond 1 to 6 weeks unless treated aggressively.^[6]^ Typically, patients with GTF and TEF complain of an irritating cough precipitated by swallowing liquids. However, the symptom is often attributed to irradiation pneumonia, epiglottis dysfunction, or tumor compression, unless the fistula is demonstrated on CT or bronchoscopy.^[7]^ Repeated lung infections, aspiration pneumonia, and severe nutritional deficiencies are the common complications of GTF and TEF, and repeated aspiration pneumonia may ultimately lead to septicemia and death.^[8]^ The fistulas do not heal spontaneously and can pose a major barrier for subsequent chemotherapy and radiation therapy.

Various new treatments such as mesenchymal stem cell transplantation^[9]^ and glue injection^[10]^ are being investigated for treatment of these fistulas but, according to the European Society of Gastrointestinal Endoscopy, covered SEMS placement remains the first choice.^[11]^ Tracheal stenting is probably the best option for high cervical GTF or TEF, as tubular esophageal stents in this location are easily displaced and are often not tolerated because of the foreign body sensation. We used a partially covered Y-shaped metallic tracheal SEMS. The covered part of the stent was used to block the fistula. The normal mucosal ciliary function at the uncovered parts decreased the possibility of sputum retention. As far as we know, this the first report of such a SEMS.

The technical success rate in our small sample was 100%. All patients experienced prompt relief of symptoms (productive cough and dyspnea) after stent placement. Even a patient with incomplete closure of the fistula experienced relief, probably because of the decrease in the entry of gastric acid and food particles into the airway.

A few previous studies have evaluated QoL in patients with GTF or TEF; most have however focused on patients with benign or malignant tracheobronchial stenosis.^[12,13]^ In the present study, the SF-36 questionnaire showed significant improvement in health-related QoL. The improvement was also reflected by the change in ECOG scores. The majority of patients (81.5%; 13/16) were able to resume oral feeding and achieve the physical fitness necessary for continuation of anticancer treatment. In the SF-36, the domains of mental health and role emotion showed no significant improvement. This is not really unexpected; while successful stenting can reduce some of the physical disabilities, it does not markedly alleviate the mental stresses associated with progressive disease. Thus, the need for psychological support should not be ignored. Biabas et al^[12]^ and Ost et al^[13]^ have previously emphasized the need for attention to both physical and mental health for long-term maintenance of QoL for patients with tracheal diseases.

In our sample, major complications occurred in 7/16 (43.8%) patients; this incidence is within the range previously reported with tracheal Y-shape stents (16.6%–51.9%).^[14,15]^ Severe tumor ingrowth (n = 2) and granulation tissue hyperplasia (n = 1) occurred even after multiple endoscopic treatments; this was not surprising, given that stenting was not accompanied by anticancer treatment. It is obvious that anti-tumor therapy should be started as soon as possible after improvement in the patient's physical condition. One patient (ECOG score = 3) in our series had massive mucostasis after stent placement and needed stent replacement after multiple endoscopic treatments failed to provide relief. The strong radial force exerted by the noncovered part of the stent can stimulate granulation tissue hyperplasia, but most of our patients had only mild hyperplasia; only 2 patients developed severe hyperplasia and required repeated endoscopic management and stent replacement. The reason for the low incidence of this complication may be that 87.5% of our patients had received external irradiation therapy and underwent timely endoscopy treatments.

Over the 2.3 to 18 months of follow-up, 7 patients died. The fact that most of them (62.5%, 10/16) had baseline ECOG score of 3 or 4 suggests ECOG score ≥3 could be a useful clinical predictor of long-term outcome; however, our sample was too small to be evaluated by multivariate regression analysis. In a retrospective analysis of 71 patients treated by SEMS in Brazil, Ribeiro et al^[16]^ found that ECOG 3 or 4 and fistula development were independent predictors of outcomes.

This study has several limitations. It was retrospective study of a very small sample, with no control group and a limited observation period. In addition, the effect of SEMS length and follow-up treatments on outcomes were not evaluated.

To conclude, the newly developed partially covered tracheal Y-shaped SEMS appears to be safe and effective for treatment of high cervical GTF and TEF. However, large randomized controlled studies are needed to confirm our findings, identify long-term complications, and optimize follow-up management.

## Author contributions

**Conceptualization:** Dechao Jiao, Yuan Yao, Jianzhuang Ren, Xinwei Han.

**Data curation:** Yuan Yao, Ling Wang.

**Formal analysis:** Dechao Jiao, Yuan Yao, Xinwei Han.

**Funding acquisition:** Dechao Jiao, Xinwei Han.

**Investigation:** Dechao Jiao, Xinwei Han.

**Methodology:** Yuan Yao.

**Project administration:** Quanhui Zhang, Ling Wang, Xinwei Han.

**Resources:** Quanhui Zhang, Ling Wang.

**Software:** Yuan Yao, Ling Wang.

**Supervision:** Quanhui Zhang, Jianzhuang Ren, Xinwei Han.

**Validation:** Yuan Yao.

**Visualization:** Jianzhuang Ren.

**Writing – original draft:** Dechao Jiao, Yuan Yao, Xinwei Han.

**Writing – review & editing:** Dechao Jiao, Quanhui Zhang, Jianzhuang Ren, Xinwei Han.
